# Exploring the Mechanism of Danshensu in the Treatment of Doxorubicin-Induced Cardiotoxicity Based on Network Pharmacology and Experimental Evaluation

**DOI:** 10.3389/fcvm.2022.827975

**Published:** 2022-02-28

**Authors:** Jia-ying Qi, Ya-kun Yang, Chuan Jiang, Yang Zhao, Yong-chao Wu, Xue Han, Xuan Jing, Zhong-lin Wu, Li Chu

**Affiliations:** ^1^School of Pharmacy, Hebei University of Chinese Medicine, Shijiazhuang, China; ^2^School of Preventive Medicine, Hebei University of Chinese Medicine, Shijiazhuang, China; ^3^Department of Radiology and Interventional Medicine, The Fourth Hospital of Hebei Medical University, Shijiazhuang, China; ^4^Affiliated Hospital, Hebei University of Chinese Medicine, Shijiazhuang, China

**Keywords:** Doxorubicin, Danshensu, network pharmacology, oxidative stress, inflammation, Keap1-Nrf2/NQO1

## Abstract

**Background:**

Doxorubicin (DOX) is one of the most effective chemotherapeutic agents available; however, its use is limited by the risk of serious cardiotoxicity. Danshensu (DSS), an active ingredient in Salvia *miltiorrhiza*, has multiple cardioprotective effects, but the effect of DSS on DOX-induced cardiotoxicity has not been reported.

**Objectives:**

Predicting the targets of DOX-induced cardiotoxicity and validating the protective effects and mechanisms of DSS.

**Methods:**

(1) Using methods based on network pharmacology, DOX-induced cardiotoxicity was analyzed by data analysis, target prediction, PPI network construction and GO analysis. (2) The cardiotoxicity model was established by continuous intraperitoneal injection of 15 mg/kg of DOX into mice for 4 days and the protective effects and mechanism were evaluated by treatment with DSS.

**Results:**

The network pharmacology results indicate that CAT, SOD, GPX1, IL-6, TNF, BAX, BCL-2, and CASP3 play an important role in this process, and Keap1 is the main target of DOX-induced cardiac oxidative stress. Then, based on the relationship between Keap1 and Nrf2, the Keap1-Nrf2/NQO1 pathway was confirmed by animal experiments. In the animal experiments, by testing the above indicators, we found that DSS effectively reduced oxidative stress, inflammation, and apoptosis in the damaged heart, and significantly alleviated the prolonged QTc interval caused by DOX. Moreover, compared with the DOX group, DSS elevated Keap1 content and inhibited Nrf2, HO-1, and NQO1.

**Conclusion:**

The results of network pharmacology studies indicated that Keap1-Nrf2/NQO1 is an important pathway leading to DOX-induced cardiotoxicity, and the results of animal experiments showed that DSS could effectively exert anti-oxidative stress, anti-inflammatory and anti-apoptotic therapeutic effects on DOX-induced cardiotoxicity by regulating the expression of Keap1-Nrf2/NQO1.

## Introduction

Doxorubicin (DOX), an anthracycline antibiotic, is the first-line drug for the clinical treatment of chemotherapy. It has been widely used to treat human tumors like leukemia, lymphoma, and solid malignant tumor (1). At the same time, the application of DOX is restricted to a certain extent due to severe heart failure and irreversible cardiomyopathy (2, 3).

There is no doubt that DOX-induced cardiotoxicity is a multifactorial process, and oxidative stress is considered to be a key factor in it (4). Reactive oxygen species (ROS) are natural byproducts of normal oxygen metabolism and have an important role in cell signaling and balancing the oxidative state *in vivo*. Mitochondria are the main organelle for ROS production, and once DOX enters the cardiomyocyte, nicotinamide adenine dinucleotide phosphate oxidase, and nitric oxide synthase in the mitochondria convert its quinine group to semiquinone. This semi-quinone reacts with O^2−^ and is converted to H_2_O_2_ by superoxide dismutase (SOD). Dangerously, H_2_O_2_ and O^2−^ have the potential to produce highly unstable and toxic hydroxyl radicals (OH^−^) in the iron-catalyzed Haber-Weiss reaction (5). High levels of ROS not only stimulate cell membrane lipid peroxidation, causing oxidative damage to mitochondria and cell membranes in cardiomyocytes, but also activate nuclear transcription factor damage and upregulate expression factors of inflammation (6). Severe oxidative stress can regulate B-cell lymphoma-2 (BCL-2) and BCL2-associated X (BAX) in mitochondria, and finally activate Caspase-3 (CASP3) to induce apoptosis (7). Studies have confirmed that DOX-induced cardiotoxicity triggers arrhythmias, including prolonged QT interval, bradycardia and ST-segment elevation (8), cardiomyopathy, left ventricular insufficiency, and congestive heart failure (9).

DOX-induced cardiotoxicity is classified as “heart failure disease” and “palpitation” in Chinese medicine (10) with palpitations and blood stasis as the main symptoms. Treatment should be based on activating blood stasis and warming the heart yang (11). Although dexrazoxane is the only drug approved by the FDA to protect against cardiotoxicity caused by anthracyclines, it still has side effects such as causing an increased incidence of second primary malignancies (12). Currently, numerous pharmacologists are searching for monomeric components of plants that can mitigate DOX-induced cardiotoxicity.

**Figure 1:**
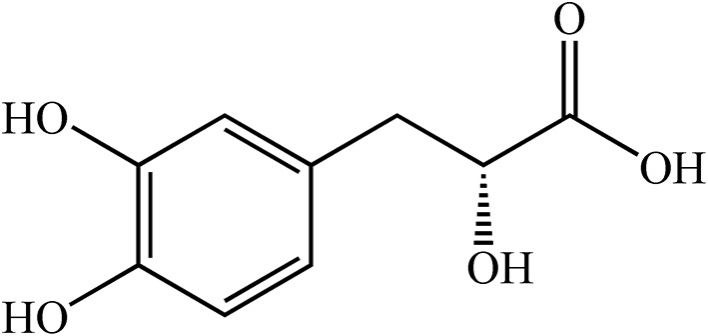
Chemical structure of Danshensu.

Danshen (*Salvia miltiorrhiza* Bge), coming from the root and rhizome of Danshen, is widely used to protect against heart diseases such as myocardial ischemia and atherosclerosis (13). Danshensu [DSS, C_9_H_10_O_5_, 3-(3, 4-dihydroxy-phenyl) lactic acid, Figure 1], is a water-soluble constituent from Salvia *miltiorrhiza* whose structure is composed of catechol and lactic acid. Catechol is the main active group that plays an antioxidant role in protecting the heart from cardiac ischemia-reperfusion injury (14). DSS has many pharmacological effects, such as anti-oxidation, anti-apoptosis, treatment of myocardial ischemia, and relief of myocardial hypertrophy (15–18). In addition, the protective effect of DSS on the heart may be due to the decrease of intracellular calcium concentration by inhibiting L-type calcium current and contraction of cardiomyocytes; it has a similar effect of calcium antagonists (19). However, the protective effect of DSS pretreatment against DOX-induced cardiotoxicity needs to be further explored. Studies have shown that treatment with angiotensin-converting enzyme inhibitors improves the prognosis of patients with chronic heart failure (20). As an angiotensin-converting enzyme inhibitor, Captopril (CAP) has been reported to be a standard protective agent (21) that significantly ameliorates DOX-induced cardiotoxicity by inhibiting oxidative stress and inflammation (22, 23). Therefore, CAP will be used as a positive control drug in this study (24).

Network pharmacology is a method of drug design, based on the rapid development of bioinformatics, systems biology, and multi pharmacology. Traditional drug design adheres to the concept of finding the most selective ligand to act on a single drug target (25). However, a large number of failed drug development trials in clinical phase 2 and 3 have taught us that multi-target synergy in disease may be the key to finding ligands (26). By matching molecular structures and screening molecules for absorption, distribution, metabolism, and excretion *in vivo*, we can obtain ligands with high correlation to disease targets (27). Three complementary approaches to constructing a disease “complex protein/gene” network are systematic screening, network analysis, and knowledge-based combinations, and this network has yielded excellent results in unraveling small molecule regulatory principles and screening for receptor affinity (25).

According to the existing reports on DOX-induced cardiotoxicity, this study utilized a network pharmacology approach to identify key targets, confirm the preventive effects of DSS in experiments and elucidate the mechanism of action, providing ideas for further experiments.

## Materials and Methods

### Data Collection and Preparation

In the Comparative Toxicogenomics Database (http://ctdbase.com/) (28), “Doxorubicin” was used as the keyword to search, and the results were obtained and exported under “Genes.” In the GeneCards database (https://www.genecards.org/) (29), we used the search term “heart damage” as the keyword, gathered the results directly and exported them. All searches were performed in the context of *Homo sapiens*.

### Network Construction and Analysis

The collected targets were used to find the intersection and imported into Cytoscape 3.7.1 to construct the network diagram. Then, the intersection targets were first introduced into DAVID (https://david.ncifcrf.gov/) (30, 31) for Gene Ontology (GO) analysis and STRING (https://string-db.org/) (32) to construct protein-protein interaction (PPI) network. “Homo sapiens” was used as the screening condition, and the minimum interaction score was set to 0.700, through which the correlation score was obtained. GO analysis is mainly used to enrich gene related functional types including biological processes (BP), molecular functions (MF). The PPI network score was analyzed by Cytoscape 3.7.1 to extract the core targets and determine the related indicators of animal experiments.

### Materials

DSS was purchased from Yuanye Bio-Technology Co., Ltd. (Shanghai, China). DOX was purchased from Hisun Pharmaceutical Co., Ltd (Zhejiang, China). Other chemical reagents needed in this experiment were purchased from Sigma Co., Ltd. (MO, USA).

### Animals

Six-week old male adult KM mice weighing 18–21 g, given from the Skbex Biotechnology Co., Ltd (Henan, China). The animals were kept under standard conditions, i.e., normal administration of feed and water, maintaining ambient temperature (20 ± 5°C) and humidity (40–60%). The handling of animals in this experiment has been authorized by the Animal Experiment Ethics Committee of Hebei University of Traditional Chinese Medicine, with the record number DWLL2020070.

### Experimental Design

**Figure 2:**
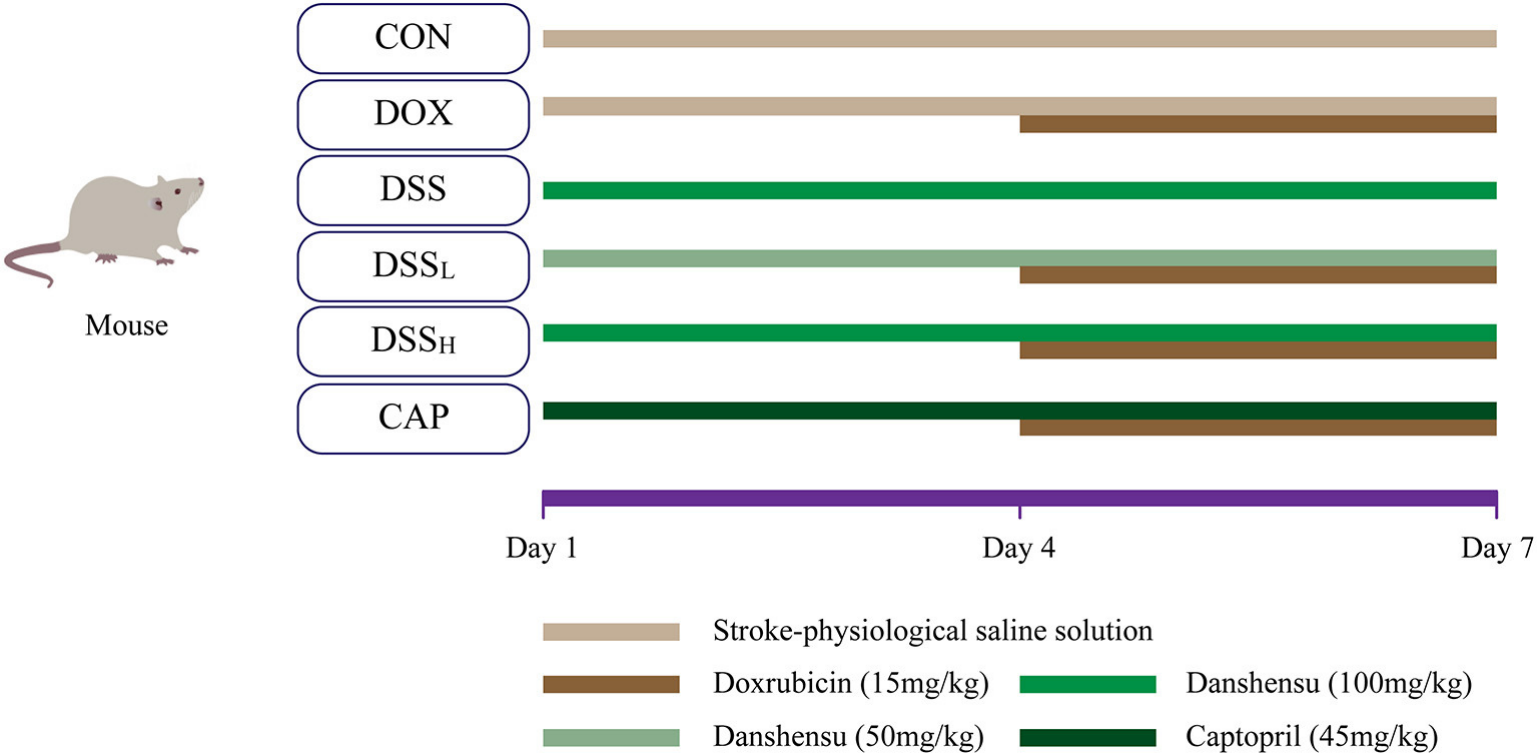
Schematic diagram of the drug delivery protocol.

After adaptive feeding for a week, and then 60 male adult KM mice were randomly divided into six groups (10 mice in each group): CON group, DOX group, DSS group, low-dose DSS + DOX group (DSS_L_), high-dose DSS+DOX group (DSS_H_), CAP+DOX group (CAP). The DOX (15 mg/kg) dose was established based on previous studies (33). The DSS_L_ and DSS_H_ groups were administrated with DSS (50, 100 mg/kg/day, i.p.), and the CAP group was administrated with CAP (45 mg/kg/day, i.p.) separately. Normal saline was given to the CON group and DOX group and corresponding doses of drugs were given to the DSS pretreatment group and CAP group for 3 consecutive days. After 4 days of pretreatment, DOX was administered 6 h after protective drug injection in days 4–7 (Figure 2) (24).

On day 8, all mice were anesthetized with sodium pentobarbital (50 mg/kg) and euthanized after ECG collection. Blood was collected from the inner canthal region, centrifuged at 3,000 r/min for 15 min, and the serum from the upper layer was drawn and stored at −20°C. Cardiac tissue was rapidly removed and part of it was stored in paraformaldehyde fixative with 0.04%, and the other was stored in liquid nitrogen.

### Electrocardiogram (ECG)

Green, black, and red subcutaneous needle electrodes were connected to the left upper limb, left lower limb, and right lower limb of the mice, respectively, and the ECGs were collected with an RM6240BD biosignal acquisition and processing system (Chengdu Instrument Co., Ltd., Chengdu, China). The QTc interval (QTc) was corrected using the Bazett formula: QTc = QT/√R-R, with R-R being the normalized heart rate value.

### Histopathological Analysis

The hearts of mice in each group were fixed in 4% paraformaldehyde for 48 h and then embedded in paraffin; the sample was processed into 3–5 μm thick sections and treated with hematoxylin-eosin (H&E) and examined under a light microscope (400×).

### Serum Biochemical Analysis

After collection, the blood of mice was centrifuged at 3,000 r/min for 10 min at 37°C. According to the instructions of the kits, determination of creatine kinase (CK) (Catalog number: A032-1-1), lactate dehydrogenase (LDH) (Catalog number: A020-2-2), SOD (Catalog number: A001-3-2), catalase (CAT) (Catalog number: A007-1-1), and Glutathione peroxidase (GSH-Px, GPX) (Catalog number: A005-1-2) activities and malondialdehyde (MDA) (Catalog number: A003-1-1) levels in serum. All kits were purchased from Jiancheng Institute of Biological Engineering (Nanjing, China).

### ROS Level Analysis

The heart tissues were gradient eluted using different concentrations of ethanol, then immersed in xylene to make the tissues transparent, and finally paraffin-embedded at 52–54°C. The sample was cut into 5 μm thick sections, and 10 μM dihydroethidium was added, and the ROS staining solution was added and incubated 30 min in the dark at 37°C. The slides were washed three times with PBS (5 min/time) before observation. A fluorescence microscope (Nikon EclipseC1, Nikon, Tokyo, Japan) was used to detect and collect images. ROS-positive cells were labeled red with dye and present the results with a light microscope (200×). The degree of cellular oxidation was quantified as the area of fluorescent staining.

### Inflammatory Factor Levels Analysis

Levels of inflammatory factors were measured by ELISA with Interleukin- 6 (IL-6) kit (MAN0017508, 88-7064) and tumor necrosis factor-α (TNF-α) kit (MAN0017423, 88-7324) purchased from Thermo Fisher Scientific (Massachusetts, America). Heart tissue was cut up, added to nine times the volume of PBS (pH = 7.4), transferred to a glass homogenizer and made into a 10% homogenate on ice, then further ground using ultrasonic fragmentation. The prepared tissue homogenate was centrifuged at 5,000 r for 5 min and the supernatant was taken as reserve. Dilute the antibody with carbonate-coated buffer to a protein content of 10 μg/ml. Antibodies, enzyme conjugates and chromogenic substrates were added according to the instructions of the kit, and the optical density value of each well was measured at 450 nm using a microplate reader after termination.

### Western Blotting

The heart tissue was washed three times with PBS, and nine times the volume of the tissue lysate was added to get the homogenate. The homogenate was placed in the ice lysate for 30 min and oscillated every 5 min to ensure the complete lysis of the tissue. The total protein (30–50 μg) was extracted from the supernatant of the lysate after centrifugation at 12,000 r/min for 10 min at 4°C. Denatured proteins were separated by 10% SDS-PAGE electrophoresis and transferred to polyvinylidene fluoride membranes, blocking 5% skim milk in Tris-buffered saline containing Tween-20 for 1 h at room temperature. Then the membranes were incubated overnight at 4°C with the following primary antibodies: BAX (Catalog: GB11690), BCL-2 (Catalog: PAA778Mu01), CASP3 (Catalog: 66470-2-lg), Kelch-like ECH-associated protein 1 (Keap1) (Catalog: 60027-1-lg), Nuclear factor-carotenoid 2 (Nrf2) (Catalog: 16396-1-ap), heme oxygenase 1 (HO-1) (Catalog: 10701-1-ap), quinone oxidoreductase 1 (NQO1) (Catalog: GB11282), and β-actin (Catalog: GB12001). After being washed three times with TBST, the membranes were incubated with HRP-conjugated secondary antibodies for 1 h at room temperature in the dark. All primary antibody dilution ratios were 1:1,000 and secondary antibody dilution ratios were 1:3,000. The grayscale value of the bands was quantified densitometrically by ImageJ analysis software. β-actin was used as normalized of the relative protein expression.

### Statistical Analysis

All quantitative data were expressed as mean ± standard error (SEM), using one-way analysis of variance (ANOVA) with Tukey's test. All data were imported into SPSS 23.0 software for analysis, and significant differences were indicated at *P* < 0.05.

## Results

### Potential DOX Targets Related to Heart Damage

**Figure 3:**
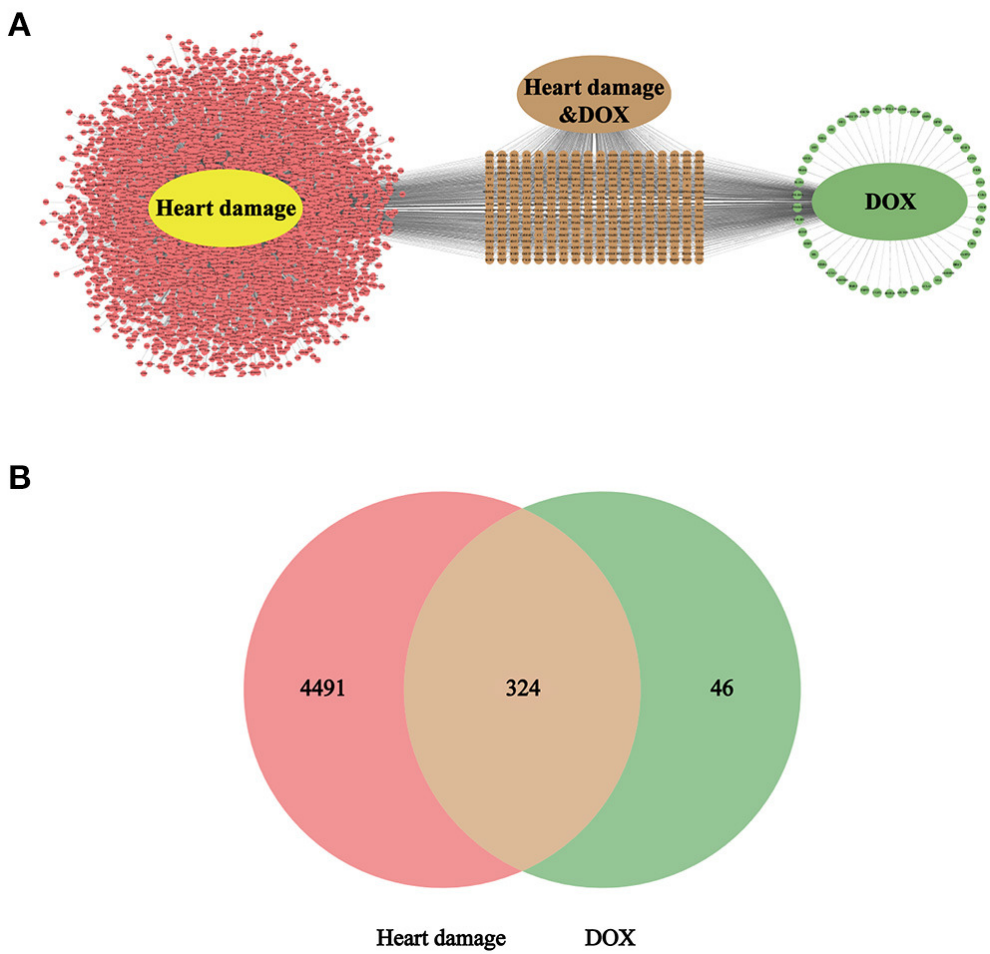
The intersection target **(A)** of heart damage and DOX and its quantitative expression **(B)**. Among them, pink represents the target of heart damage, green represents the target of DOX, and brown represents its intersection.

According to the retrieval, there are 13,984 targets in the heart damage and 8058 targets in the DOX; control the accuracy and quantity of targets, heart damage and DOX targets were screened by correlation score ≥ 3 and interaction count ≥ 5, respectively. Finally, there are 4,815 targets in the heart damage part and 370 targets in the DOX part (Figure 3A), among which there were 324 identical targets (Figure 3B).

### GO Analysis and Construction of PPI Network

**Figure 4:**
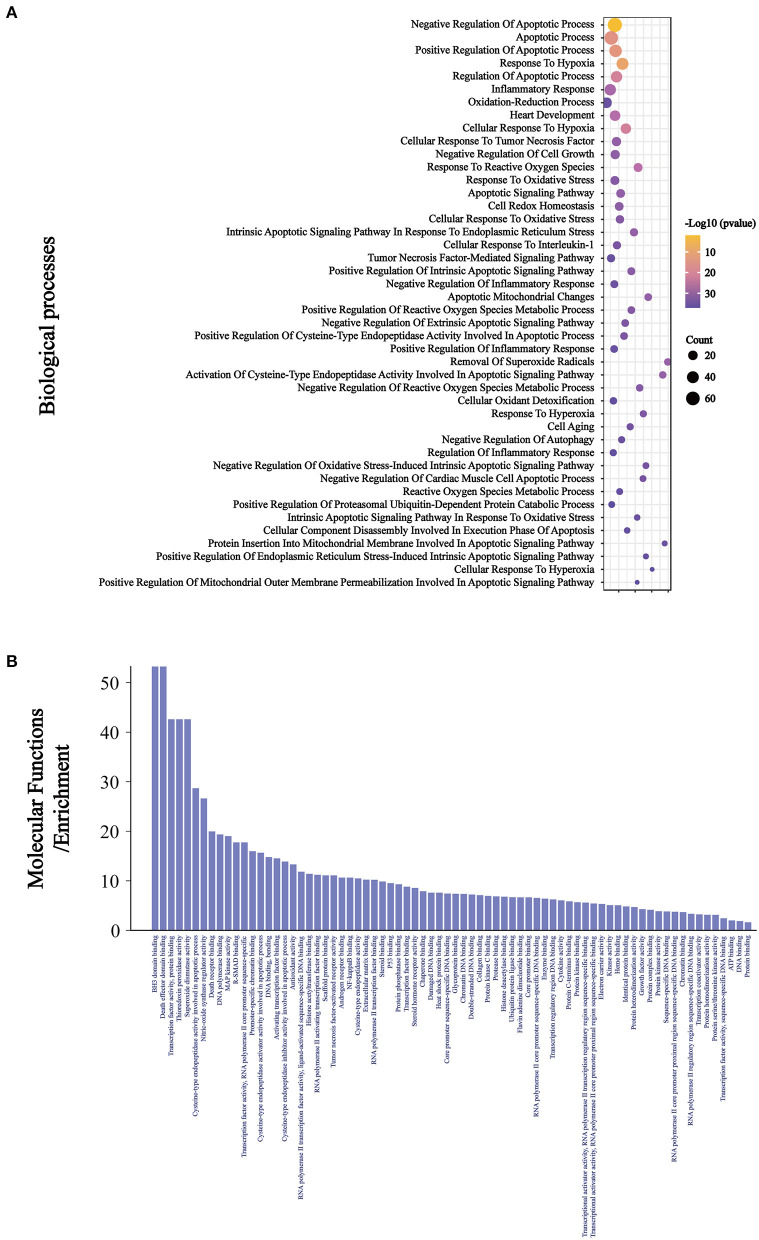
The BP **(A)** related to oxidative stress, inflammation, and apoptosis were ranked according to the number of related targets and all the MF **(B)**.

The intersection targets were imported into DAVID for GO analysis. We found that among the 401 BP, there were 15 related to oxidative stress, 18 related to apoptosis, six related to inflammation, and the other four were heart development, negative regulation of cell growth, cell aging, and negative regulation of autophagy (Figure 4A). Among the 75 MF, ubiquitin protein ligase binding, NF-κB binding, antioxidant activity, and tumor necrosis factor activated receptor activity were related to Keap1, inflammation, oxidative stress, and apoptosis (Figure 4B).

**Figure 5:**
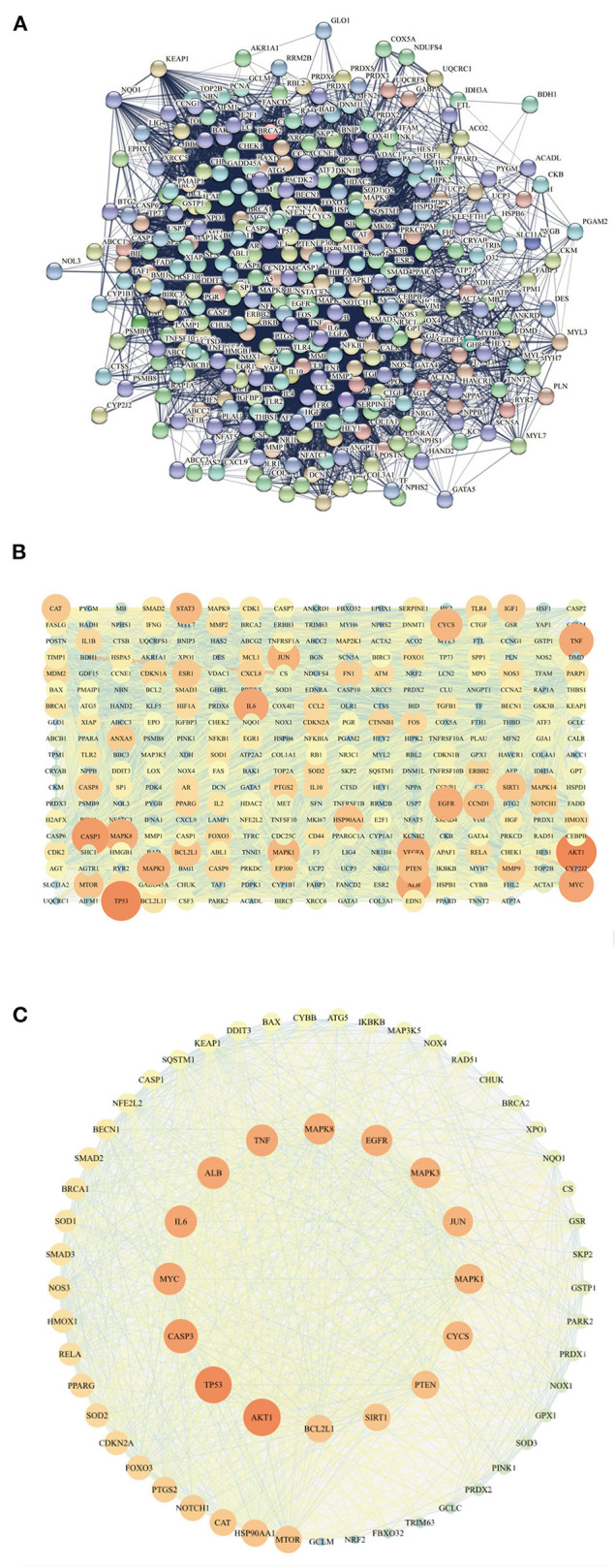
The PPI network **(A)** of intersecting targets is represented in **(B)**, with color as the variable, and the size of the points is related to the degree value. The closer the color of the points and edges, the greater the importance, and the closer the color of the points and edges, the smaller the importance. All the targets related to Keap1 are sorted by the degree value **(C)**.

The intersection targets were imported into STRING (Figure 5A), and the results were imported into Cytoscape3.7.1. The constructed network graph had 321 points and 9,429 edges (Figure 5B). In GO analysis, the relationship between ubiquitin protein ligase binding and Keap1 was the key to investigating anti DOX-induced myocardial oxidative damage. Therefore, Keap1 related targets were extracted into a new network (Figure 5C), which had 65 nodes and 1,121 edges. We can see that SOD, CAT, and GPX1 are related to oxidative stress, CASP3, BCL2, CASP1, and BAX are related to apoptosis, TNF and IL-6 are related to inflammation, and Keap1, Nrf2, and NQO1 are targets of the Keap1-Nrf2/NQO1 pathway.

### Effects of DSS on Body Weight, Food, and Water Consumption

During 7 days of rearing, final body weight, food consumption, and water consumption were 19.9, 19.5, and 13.7% lower in the DOX group than in the CON group, respectively (Table 1, *P* < 0.01). These indexes were improved in the DSS treatment group (*P* < 0.01, *P* < 0.05), and there was no mortality in all groups. It has been confirmed in the autopsy that most mice in the DOX group had ascites of varying degrees. However, there was a dose-dependent reduction in ascites in the DSS treatment group. There was no significant difference between the CON group and the DSS group.

**Table 1:** Summary of changes in body weight and consumption of feed and water in mice.

**General observation**	**CON**	**DOX**	**DSS**	**DSS_***L***_**	**DSS_***H***_**	**CAP**
Initial body weight (g)	23.42 ± 0.45	23.04 ± 0.35	22.89 ± 0.56	22.63 ± 0.44	22.68 ± 0.42	23.73 ± 0.35
Final body weight (g)	30.17 ± 0.30	24.30 ± 0.36**	28.22 ± 0.38	25.71 ± 0.28^##^	26.19 ± 0.27^##^	28.20 ± 0.54^##^
Mean food consumption (g/mice/day)	6.11 ± 0.15	4.96 ± 0.27**	5.92 ± 0.11	5.46 ± 0.13	5.57 ± 0.12	5.65 ± 0.13^#^
Mean water consumption (mL/mice/day)	3.86 ± 0.05	3.33 ± 0.25	3.89 ± 0.05	3.51 ± 0.13	3.90 ± 0.10^#^	3.74 ± 0.05
Mortality (ratio %)	0.00	0.00	0.00	0.00	0.00	0.00

*Values are given as mean ± SEM (n = 10 and 7) or as ratio*.

** *P < 0.01 vs. CON*;

# *P < 0.05; and*

## *P < 0.01 vs. DOX*.

### Effects of DSS on ECG

**Figure 6:**
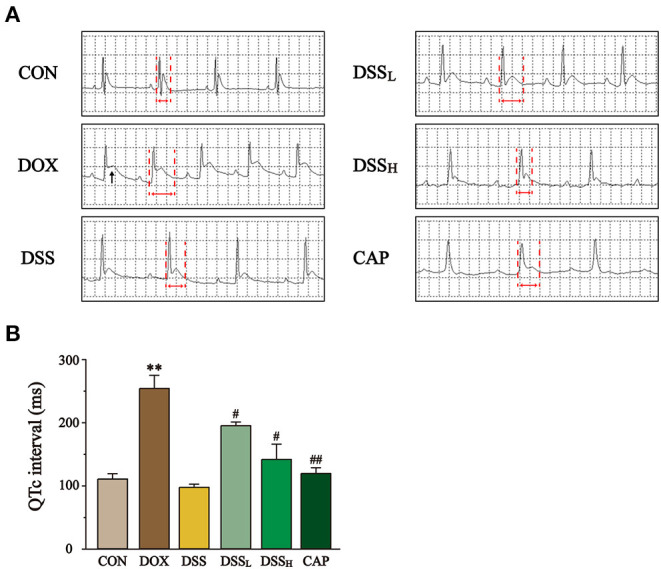
Effect of DSS on the ECG of DOX-induced cardiac injury in mice. **(A)** Representative electrocardiograms of each group. Black arrows mark ST-segment elevation and red arrows mark QT interval. **(B)** Statistics of QTc interval for each group. Data are presented as the mean ± SEM (*n* = 3). **P < 0.01 vs. CON; ^#^*P* < 0.05; and ^##^*P* < 0.01 vs. DOX.

The ECG results showed that the QTc interval in the DOX group was 2.3 times longer than that in the CON group, accompanied by significant ST-segment elevation (Figure 6) (*P* < 0.01). In contrast, compared with the DOX group, DSS pre-protection effectively alleviated the prolongation of QTc interval, which was 23.3 and 44.3% shorter in the DSS_L_ and DSS_H_ groups, respectively (*P* < 0.05). This suggests that DSS pre-protection can attenuate DOX-induced cardiac injury.

### Effects of DSS on Cardiomyocyte Morphology in DOX-Induced Cardiotoxicity

**Figure 7:**
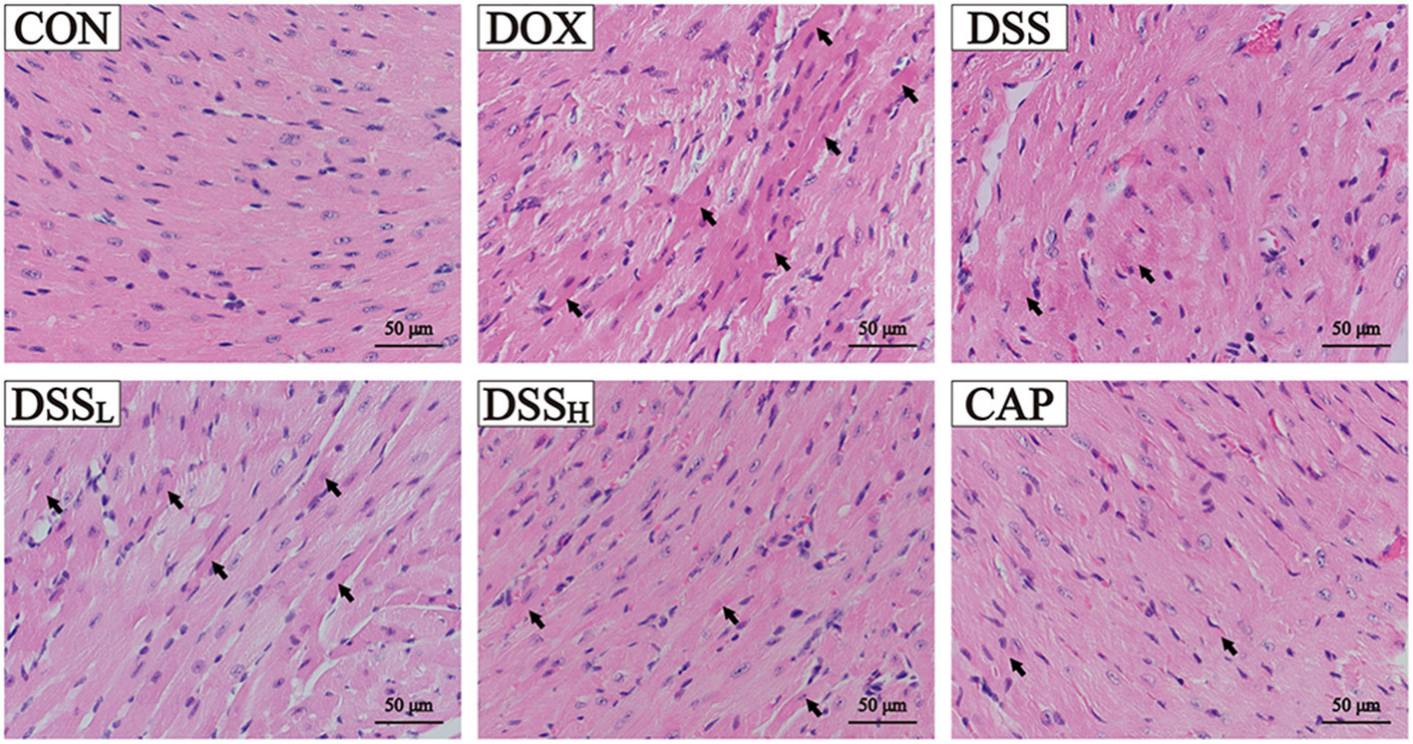
Effects of DSS on pathological changes of cardiac tissue as surveyed by H&E staining. DSS on H&E staining in DOX-induced cardiotoxicity in mice. Scale bar = 50 μm (400×).

In Figure 7, myocardial tissue sections of the DOX group showed a large number of necrotic cardiomyocytes and obvious nuclear lysis. However, the extent of myocardial injury in the DSS-treated group was distinctly lower than that in the DOX group. Especially in the DSS_H_ group, where most of the cells remained structurally normal with clear transverse lines and only a few necrotic myocardial cells were present. The pathological results indicated that the DSS-treated group showed a dose-dependent reduction of cardiac injury. Both the CON and DSS groups showed normal cardiomyocyte morphology and were not significantly different.

### Effects of DSS on Injury in Heart

**Figure 8:**
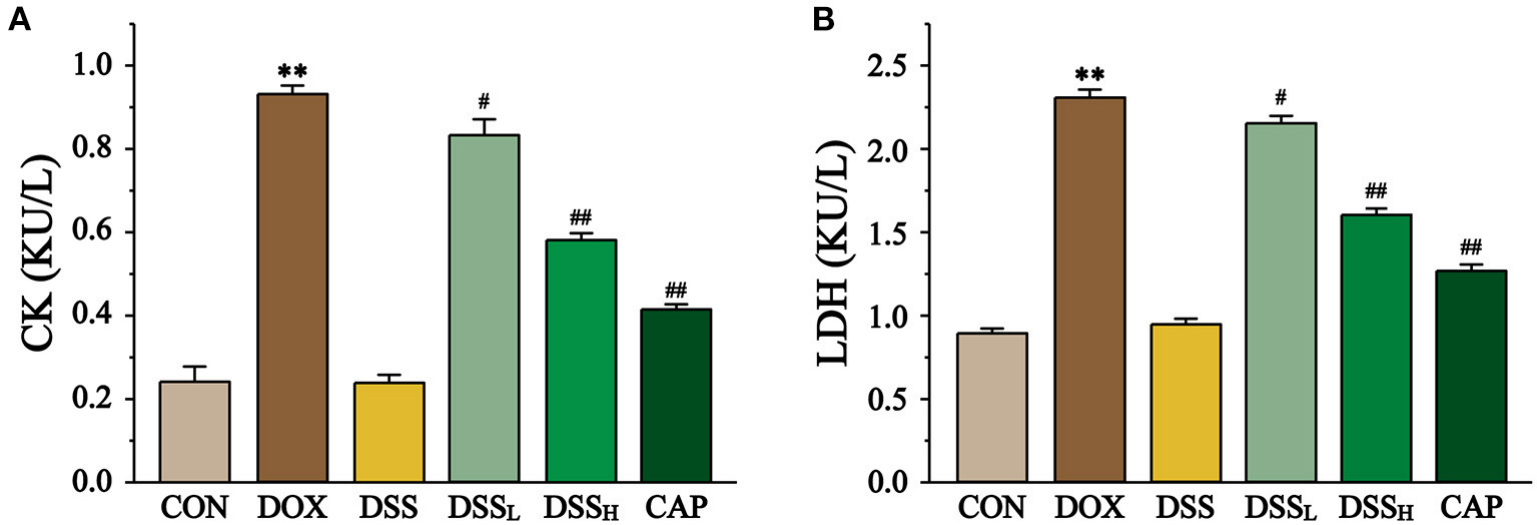
Effect of DSS on CK **(A)** and LDH **(B)** activities in DOX-induced cardiotoxicity in mice. Serum was collected from mice hearts of the CON, DOX, CAP, DSS_L_, DSS_H_, and DSS. Data are presented as the mean ± SEM (*n* = 10). **P < 0.01 vs. CON; ^#^*P* < 0.05; and ^##^*P* < 0.01 vs. DOX.

As the sign enzymes of heart injury, DOX group CK and LDH activities were increased 2.9-fold and 1.6-fold compared with the CON group, respectively (Figure 8, *P* < 0.01). Compared with the DOX group, DSS_L_ reduced CK and LDH by 10.6 and 6.7%, DSS_H_ reduced CK and LDH by 37.9 and 30.4%, respectively (*P* < 0.01). There was no significant difference between the CON group and the DSS group.

### Effects of DSS on ROS and Oxidative Stress

**Figure 9:**
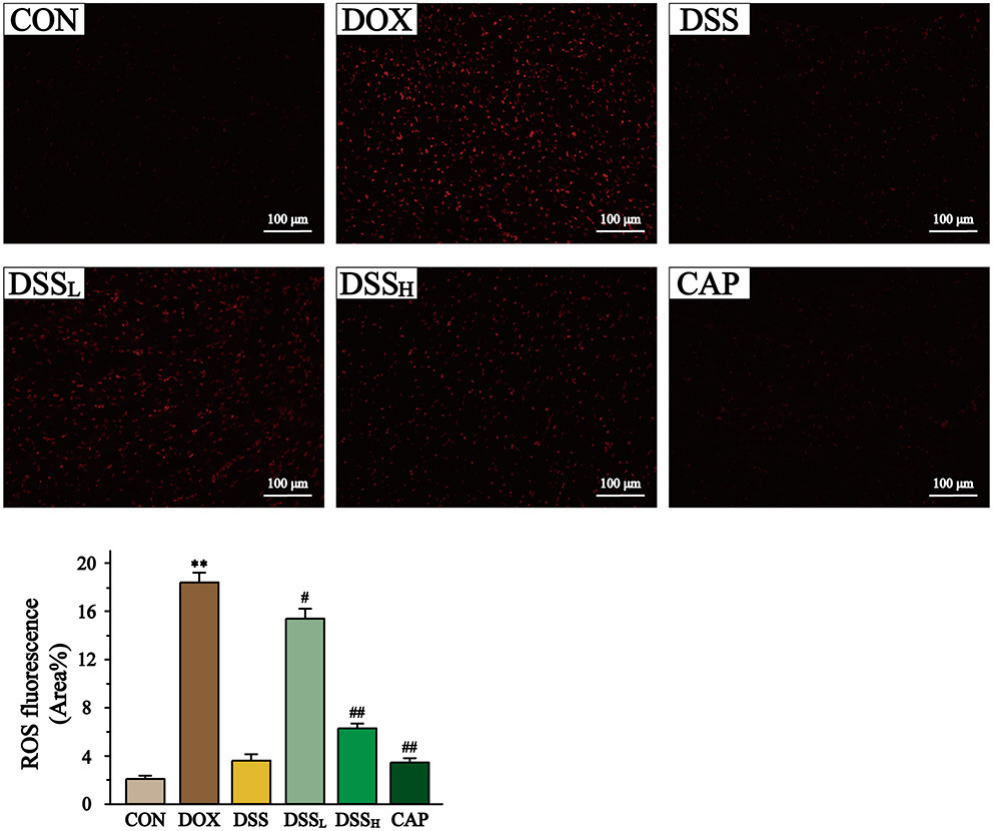
Effect of DSS on the level of ROS in myocardial tissue. Scale bar = 100 μm (200×). The fluorescence intensity and area were expressed as a percentage. Statistics of ROS fluorescent area. Data are presented as the mean ± SEM (*n* = 6). **P < 0.01 vs. CON; ^#^*P* < 0.05; and ^##^*P* < 0.01 vs. DOX.

In Figure 9, ROS content was quantified as fluorescence staining area. The ROS content in DOX group was 8.9 times higher than that in CON group (*P* < 0.01). And compared to the DOX group, the ROS content was reduced by 16.3 and 65.8% in the DSS_L_ and DSS_H_ groups, respectively (*P* < 0.01). There was no significant difference between the CON and DSS groups.

**Figure 10:**
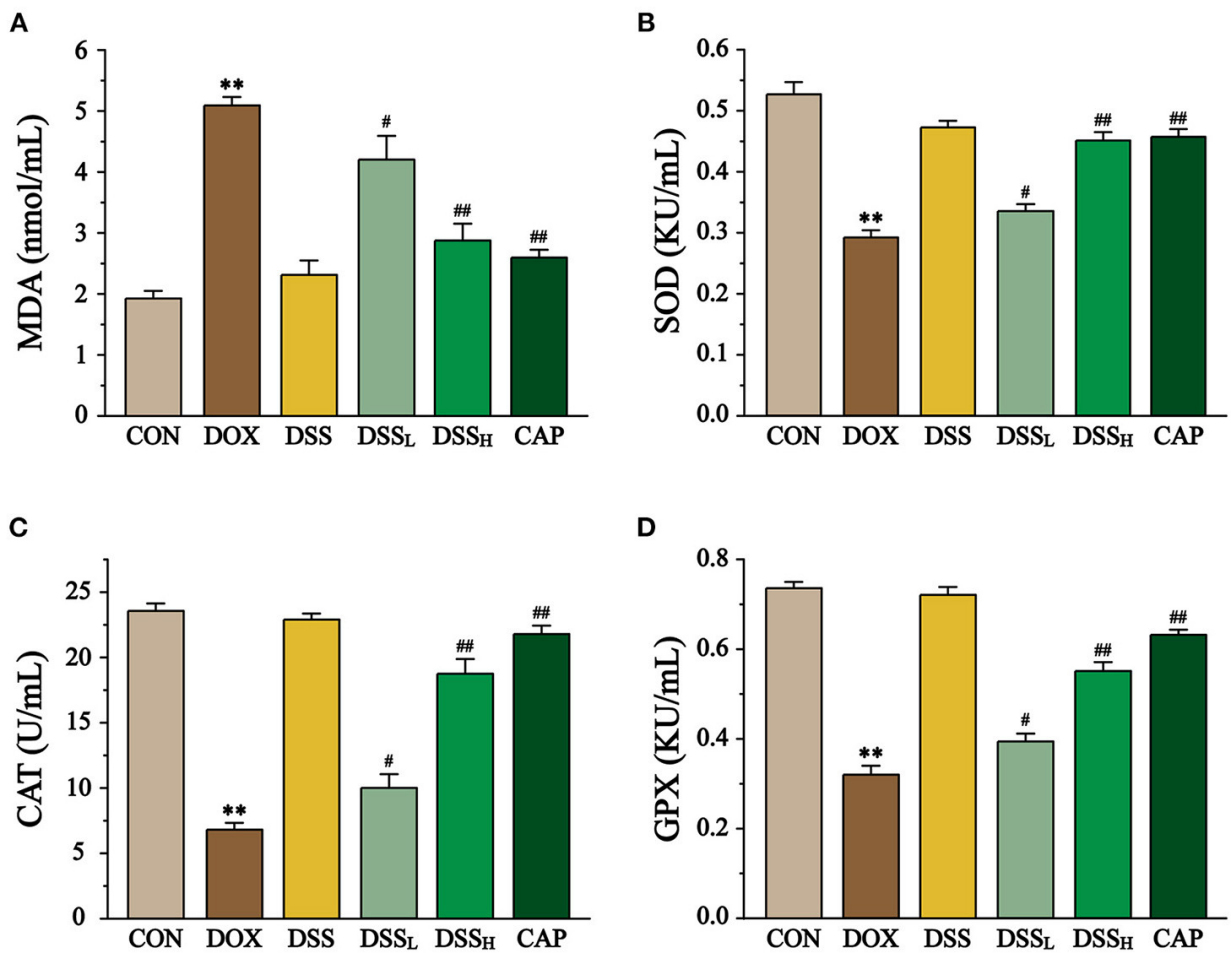
Effect of DSS on MDA content **(A)**, SOD **(B)**, CAT **(C)**, GPX **(D)** activities in DOX-induced cardiotoxicity in mice. Serum was collected from mice of the CON, DOX, DSS, DSS_L_, DSS_H_, and CAP. Data are presented as the mean ± SEM (*n* = 10, *n*=10). **P < 0.01 vs. CON; ^#^*P* < 0.05; and ^##^*P* < 0.01 vs. DOX.

Compared to the CON group, the DOX group showed a 1.6-fold increase in MDA concentration, while the DSS_L_ and DSS_H_ groups significantly downregulated MDA concentration (17.9 and 43.6%, respectively) (Figure 10, *P* < 0.01, *P* < 0.05). In the DOX group, the activities of SOD, CAT, and GPX were significantly downregulated (55.4, 71.1, and 56.6%, respectively). All of the above indicators were inversely regulated compared to the DOX group, with the DSS_L_ and DSS_H_ groups upregulating SOD by 15.5 and 54.4%, CAT by 46.7 and 175.4%, and GPX by 23.1 and 72.3%, respectively (*P* < 0.01, *P* < 0.05). There was no significant difference between the CON and DSS groups.

### Effects of DSS on IL-6, TNF-α in Heart

**Figure 11:**
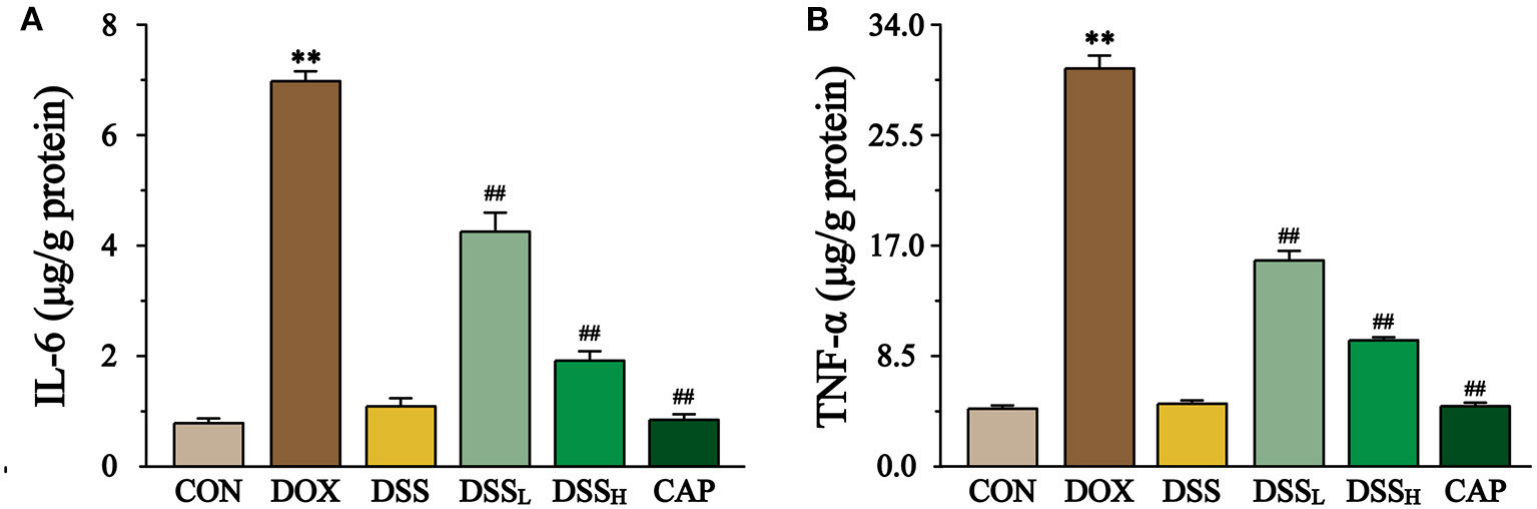
Effect of DSS on the levels of IL-6 **(A)** and TNF-α **(B)**. Data are presented as the mean ± SEM (*n* = 6). **P < 0.01 vs. CON; ^##^*P* < 0.01 vs. DOX.

The content of IL-6, TNF-α were measured to indicate the degree of inflammation of the heart by ELISA (Figure 11). The results showed that the levels of TNF-α and IL-6 in the DOX group were 6.9 and 8.9 times higher than CON group, whereas the levels of IL-6 were down-regulated by 39.1 and 72.6%, and the levels of TNF-α by 48.2 and 68.4% in the DSS_L_ and DSS_H_ groups, respectively (*P* < 0.01). There was no significant difference between the CON group and the DSS group.

### Effects of DSS on the Expression of BAX, BCL-2, CASP3

**Figure 12:**
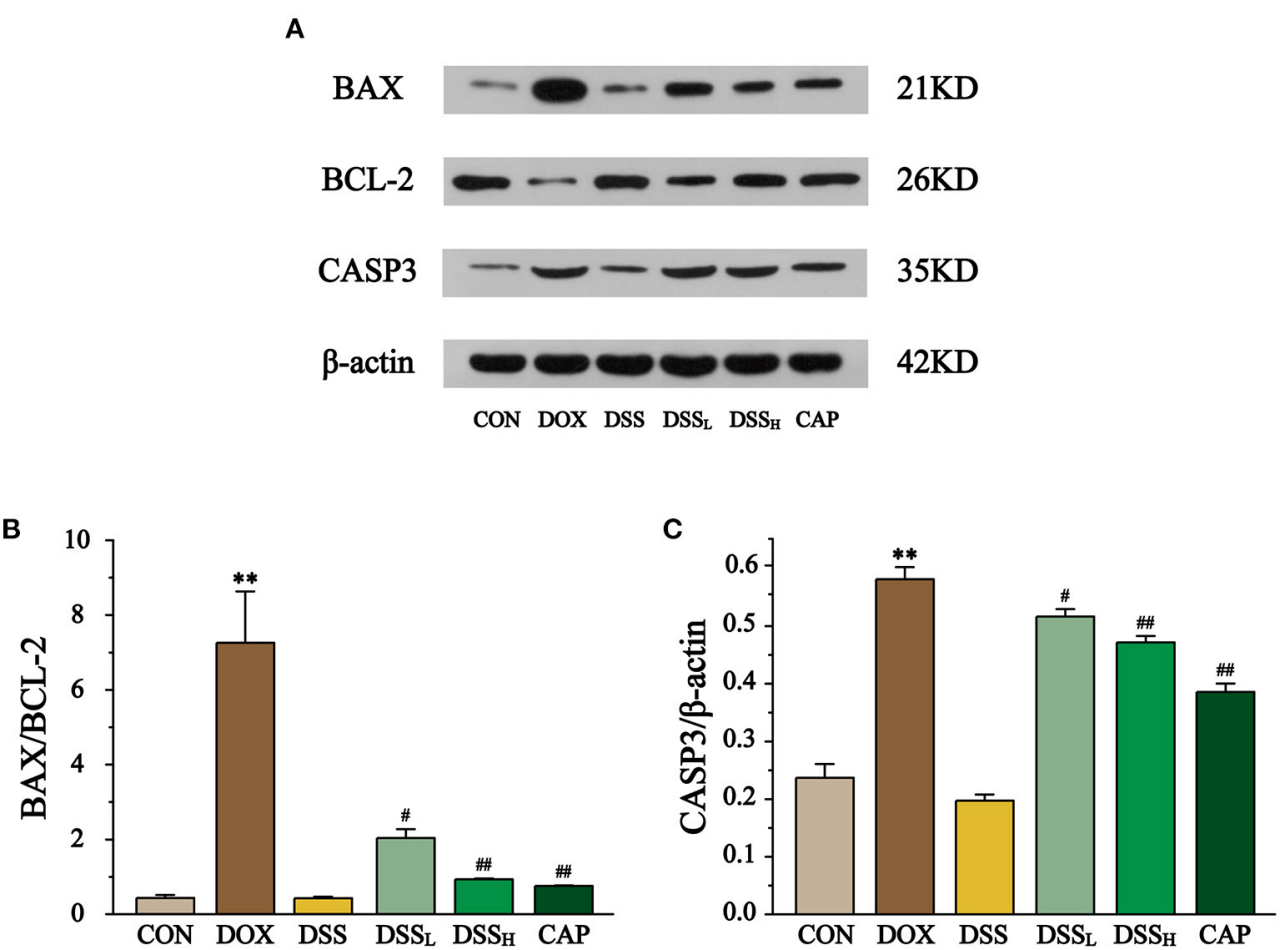
Effect of DSS on the protein expressions of BAX, BCL-2, and CASP3 **(A)**. The band intensities of BAX/BCL-2 **(B)** and CASP3 **(C)** were normalized to β-actin. Data are presented as the mean ± SEM (*n* = 3). **P < 0.01 vs. CON; ^#^*P* < 0.05; and ^##^*P* < 0.01 vs. DOX.

The effect of DSS on the expression of BAX, BCL-2, and CASP3 in myocardial tissue was detected by Western blotting (Figure 12). The results showed that BAX/BCL2 was 16.8-fold higher in the DOX group than CON group, but compared with the DOX group, BAX/BCL-2 was down-regulated by 72.0 and 87.2% in the DSS_L_ and DSS_H_ groups, respectively (*P* < 0.01, *P* < 0.05). Meanwhile, CASP3 expression was 2.5-fold higher in the DOX group than CON group, but compared with the DOX group, CASP3 expression was down-regulated by 11.1 and 18.8% in the DSS_L_ and DSS_H_ groups, respectively (*P* < 0.01, *P* < 0.05). There was no significant difference between the CON group and the DSS group.

### Effects of DSS on the Expression of Keap1, Nrf2, HO-1, and NQO1

**Figure 13:**
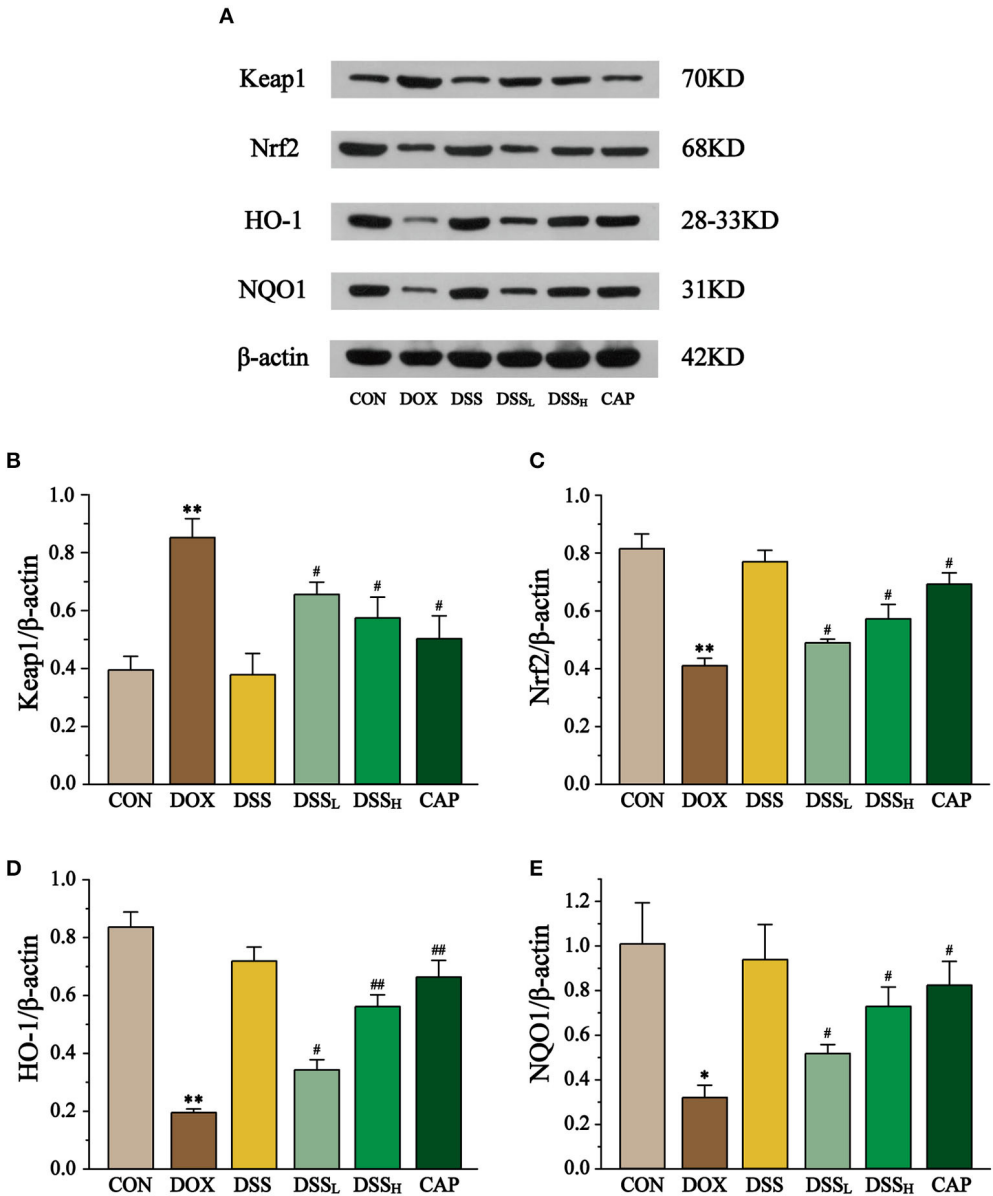
Effect of DSS on the expression of Keap1, Nrf2, HO-1, and NQO1 **(A)**. The band intensities of Keap1 **(B)**, Nrf2 **(C)**, HO-1 **(D)**, and NQO1 **(E)** were normalized to β-actin. Data are presented as the mean ± SEM (*n* = 3). *P < 0.05 and **P < 0.01 vs. CON; ^#^*P* < 0.05; and ^##^*P* < 0.01 vs. DOX.

To explore the role of the Keap1-Nrf2/NQO1 pathway in cardiotoxicity induced by DSS in the treatment of DOX, the expression of Keap1, Nrf2, and its downstream factors HO-1 and NQO1 was determined by Western blotting (Figure 13). The results showed that the expression of Keap1 was up to 2.16-fold in the DOX group compared with the CON group, while it was down-regulated by 23 and 32.6% in the DSS_L_ and DSS_H_ groups, respectively (*P* < 0.01, *P* < 0.05). Meanwhile, DOX significantly reduced the expression of Nrf2, HO-1, and NQO1 by 50.4, 76.7, 68.3%, respectively (*P* < 0.01, *P* < 0.05). Compared with the DOX group, the expression of Nrf2 was up-regulated by 19.2 and 39.5%, HO-1 by 76 and 188.2%, and NQO1 by 62.9 and 128.0% in the DSS_L_ and DSS_H_ groups, respectively (*P* < 0.01, *P* < 0.05). There was no significant difference between the CON group and the DSS group.

## Discussion

DOX has a broad anti-cancer spectrum and strong anti-tumor activity, kill tumor cells that are in various growth cycles. The major adverse drug reactions it exhibits are leukopenia and thrombocytopenia and cardiotoxicity, therefore, its long-term use is limited (34). This study used a combination of network pharmacology and animal experiments. Network pharmacology was used to predict the targets of DOX-induced cardiotoxicity, and the core targets were searched and identified by constructing PPI networks from the intersecting targets, and the core mechanisms explored in this study were identified by GO analysis.

GO analysis clearly shows that ubiquitin protein degradation is directly related to Keap1, while Nrf2 and NQO1 are binding and downstream proteins of Keap1. The keap1-Nrf2 pathway is the essential endogenous anti-oxidative stress pathway with a memorable role in reducing the extent of cardiac injury (35, 36). In summary, the Keap1-Nrf2/NQO1 pathway is the focus of this study in terms of animal experiments.

Keap1 is a major intracellular regulator of Nrf2, and Keap1-Nrf2 is normally bound and relatively stable anchored in the cytoplasm to play an important role in the cellular antioxidant stress system (37). Nrf2 negatively regulates oxidative stress in cells; there are seven highly conserved domains named Neh1–7 (38). Keap1 binds to the Neh2 domain of Nrf2 and promotes the ubiquitination and degradation of Nrf2 in the cytoplasm by E3 ubiquitin ligase containing CUL3 under normal conditions (39). The cysteine residues of Keap1 are oxidized when cells are exposed to oxidative stress, leading to the dissociation of Nrf2 from the Keap1-Nrf2 complex. Free Nrf2 is transferred to the nucleus and binds to small Maf transcription factors through the Neh1 domain (40). The Maf protein belongs to the basic leucine zipper (BZIP) family, which is mainly located in the nucleus and interacts with DNA. Nrf2/Small Maf binds to a cis-acting enhancer sequence known as the antioxidant response element (ARE) to control basal and inducible expression of downstream antioxidant and detoxification genes (41).

Consequently, Nrf2 enhances the antioxidant capacity of cells by enhancing the expression of detoxifying enzymes such as HO-1 and epoxide hydrolase (42). HO-1 and NQO1, as the downstream factors of Nrf2 activation, have been considered important intracellular antioxidants against intracellular and extracellular oxidative stress (43). Deletion or repressed expression of Nrf2 leads to abnormal remodeling of the myocardium, increased oxidative stress, apoptosis, and fibrosis (44). Nrf2 expression was found to be downregulated in DOX-treated mouse hearts and recovered after treatment to reduce DOX damage to the heart (45). These findings elucidated that Nrf2 may be a guiding drug target for the treatment of cardiotoxicity caused by DOX.

Many of myocardial injuries, such as cardiomyocyte atrophy, oxidative stress, apoptosis, and necrosis have been widely reported in experimental animals treated with DOX (46). Therefore, it is urgent to find a suitable protective agent. In the current study, we investigated the effects of DSS on oxidative stress, apoptosis, and inflammation production through pre-protection by establishing a DOX-induced cardiotoxicity model in mice, and verified whether these effects were related to the regulation of the Keap1-Nrf2/NQO1 pathway by measuring protein expression.

The weight loss and the reduction of drinking water and diet of DOX treatment mice were not found in mice pre-protected with DSS in this experiment. ECG results showed that DOX caused significant QTc interval prolongation and ST-segment elevation in the heart, indicating that the model of this study was successfully established. In addition, histopathological assessment plays an important role in confirming cardiotoxicity induced by DOX (47). These results show obvious apoptosis and nucleolytic in cardiac myocytes were found in the DOX group, whereas in the DSS pretreatment group, many intact cell structures and clear muscle striations could be observed under the microscope. This indicates that the pathological injury of the DSS group is slighter than that of the DOX group, confirming the protective effect of DSS pretreatment on DOX-induced cardiac injury.

CK and LDH are specific serum marker enzymes in the heart that would exudate when the myocardial tissue is damaged, and thus test results can indicate the degree of cardiotoxicity (48, 49). We found that the release of CK and LDH increased significantly in the DOX treatment group, but decreased significantly in the DSS pretreatment group (*P* < 0.01). The reduction of myocardial marker enzymes confirmed that DSS pretreatment improved the cardiac function of mice.

ROS is a series of reactive oxygen groups produced by aerobic cells during metabolism. DOX leads to the accumulation of many free radicals through the redox cycle, which eventually leads to the activation of reactive oxygen species. The release of a large amount of ROS is one of the main reasons for cardiotoxicity caused by DOX (50). The results of ROS fluorescence staining in this study showed that the content of ROS in the DOX treatment group was significantly higher than that in the CON group, but remarkably decreased in the DSS treatment group (*P* < 0.01). This indicates that pre-protection with DSS can effectively reduce the release of ROS in the damaged heart.

Network pharmacology analysis indicates that CAT, SOD, GPX, IL-6, TNF, BAX, BCL-2, and CASP3 are important targets of DOX-induced cardiotoxicity. As endogenous antioxidant enzymes, CAT and SOD catalyze the inactivation of ROS and are the first line of cellular defense against oxidative stress. GPX, released by GSH, can reduce peroxide H_2_O_2_ to O^−^ and H_2_O^−^ (24). ROS and other peroxides, if not removed in time, can damage the cellular phospholipid layer and cause membrane lipid peroxidation (51) and the accumulation of product MDA will lead to membrane dysfunction and membrane binding enzyme failure and other consequences (52). The results showed a substantial increase in the lipid peroxidation product MDA and a significant decrease in the activities of the antioxidant enzymes CAT, SOD, and GPX in the DOX group (*P* < 0.01), this finding is consistent with previous studies (53). We found that pre-protection with DSS effectively reduced the level of MDA and significantly increased the activities of antioxidant enzymes CAT, SOD, and GPX (*P* < 0.01). The results in this section elucidate that the remarkable antioxidant effect of DSS is an essential mechanism to attenuate DOX-induced cardiotoxicity.

It was demonstrated that oxidative stress and inflammation are inextricably linked to the progression of the disease (54). Massive accumulation of ROS further activates the NF-κB pathway and releases pro-inflammatory factors such as TNF-α and IL-6 (55), triggering a series of inflammatory reactions (56). These inflammatory reactions can lead to cardiomyopathy, transmural myocarditis, and biventricular fibrosis (57). The levels of TNF-α and IL-6 are down-regulated by pre-protection with DSS, suggesting that DSS may mitigate DOX-induced cardiotoxicity by exerting anti-inflammatory effects.

Apoptosis is also an important mechanism leading to heart injury by DOX. On the one hand, the production of reactive oxygen species triggers the apoptotic response by activating apoptosis signal-regulated kinase 1 signal (58). On the other hand, DOX causes mitochondrial dysfunction, increases mitochondrial membrane permeability, induces cytochrome release and activation of caspases (59). As pro-apoptotic and anti-apoptotic members, BAX and BCL-2 play an important role in regulating the mitochondrial apoptotic program (60). Mitochondrial membranes are the main targets of BAX and BCL-2, with the former preventing membrane permeability changes and the latter promoting mitochondrial membrane permeability and cytochrome release. They are antagonistic to each other in maintaining mitochondrial membrane permeability (61). Our results showed that DSS pretreatment effectively prevents the up-regulation of BAX and the down-regulation of BCL-2 (Figure 12), two key factors that lead to the activation and hydrolysis of downstream caspase, triggering an apoptotic cascade response (62). Caspases promoters connect downstream caspases effectors, leading to activation of the caspase family, which plays a central role in mediated apoptotic responses (63). CASP3 belongs to the caspase family, which is a critical effector for the apoptotic response and responsible for the final step of the caspase family leading to apoptosis (64–66). Several studies have shown that DOX-induced apoptosis in cardiomyocytes exhibits elevated expression of BAX/BCL-2 and CASP3 (53, 67, 68). In our study, BAX/BCL-2 and CASP3 levels in DOX group were significantly higher compared to CON group, whereas DSS pretreatment was effective in reducing them, this suggests that DSS pre-protection alleviates DOX-induced apoptosis in cardiac myocytes.

In the present research, treatment with DOX significantly up-regulate Keap1 and down-regulated Nrf2, HO-1, and NQO1. Therefore, we confirmed that Nrf2 is released to activate antioxidant function against severe cardiac injury in DOX-induced cardiotoxicity (*P* < 0.01 and *P* < 0.05). On the contrary, DSS down-regulated Keap1 in a dose-dependent manner and up-regulated Nrf2, HO-1, and NQO1 (*P* < 0.01 and *P* < 0.05). This indicates that the oxidative stress injury of the heart is weakened and the released Nrf2 is sufficient to activate the subsequent antioxidant process. The results suggest that pre-protection by DSS attenuates DOX-induced oxidative stress in the heart, which may be achieved by activating the Keap1-Nrf2/NQO1 pathway.

## Conclusion

**Figure 14:**
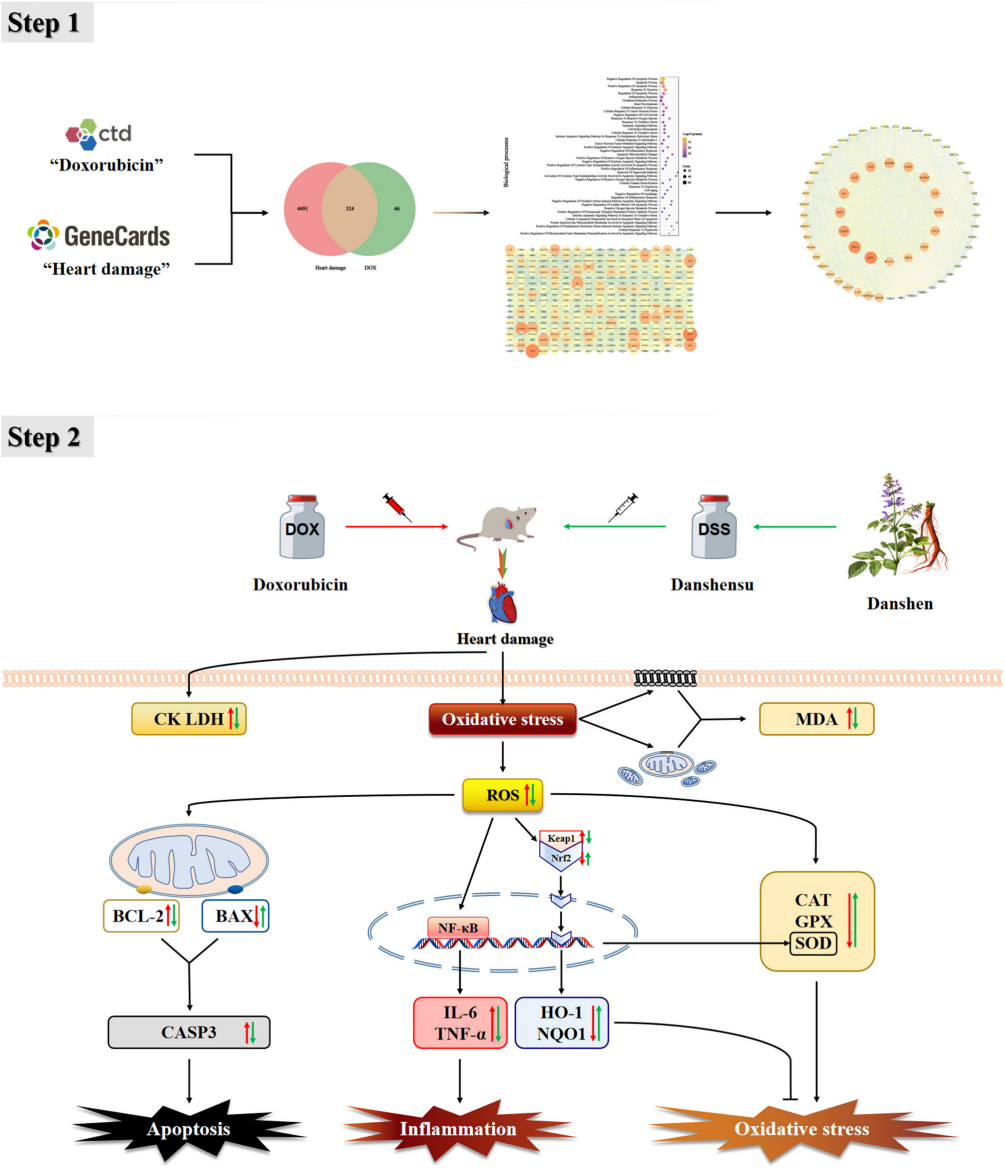
Protective mechanism of DSS against DOX-induced heart damage.

This study used network pharmacology to preliminarily confirm that CAT, SOD, GPX, IL-6, TNF, BAX, BCL-2, and CASP3 are important targets of DOX-induced cardiotoxicity, and Keap1-Nrf2/NQO1 is one of the important pathways. In animal experiments, pre-protection by DSS was shown to reduce apoptosis of cardiomyocytes and inhibit oxidative stress and inflammation, all of which occur through regulation of the Keap1-Nrf2/NQO1 signaling pathway (Figure 14). Our study indicates that Keap1-Nrf2/NQO1 is an essential pathway for DOX-induced cardiac injury, and DSS can effectively resist oxidative stress, inflammation, and apoptosis, making it a promising cardiotoxic protective agent.

## Data Availability Statement

The original contributions presented in the study are included in the article/supplementary material, further inquiries can be directed to the corresponding author/s.

## Ethics Statement

The handling of animals in this experiment has been authorized by the Animal Experiment Ethics Committee of Hebei University of Traditional Chinese Medicine, with the record number DWLL2020070.

## Author Contributions

J-yQ, Y-kY, and LC conceived and designed the experiments and wrote the manuscript. J-yQ, Y-kY, CJ, YZ, and Y-cW performed all the experiments. XH and XJ analyzed the data. Z-lW and LC engaged in material support for obtained funding and supervised the study. All authors have read and approved the final manuscript.

## Funding

This work was supported by the Research Foundation of Administration of Traditional Chinese Medicine of Hebei Province, China (No. 2019135).

## Conflict of Interest

The authors declare that the research was conducted in the absence of any commercial or financial relationships that could be construed as a potential conflict of interest.

## Publisher's Note

All claims expressed in this article are solely those of the authors and do not necessarily represent those of their affiliated organizations, or those of the publisher, the editors and the reviewers. Any product that may be evaluated in this article, or claim that may be made by its manufacturer, is not guaranteed or endorsed by the publisher.

## References

[1] Carvalho FS Burgeiro A Garcia R Moreno AJ Carvalho RA Oliveira PJ. Doxorubicin-induced cardiotoxicity: from bioenergetic failure and cell death to cardiomyopathy. Med Res Rev. (2014) 34:106–35. 10.1002/med.21280 23494977

[2] Loncar-Turukalo T Vasic M Tasic T Mijatovic G Glumac S Bajic D. Heart rate dynamics in doxorubicin-induced cardiomyopathy. Physiol Meas. (2015) 36:727–39. 10.1088/0967-3334/36/4/727 25798626

[3] Cardinale D Iacopo F Cipolla CM. Cardiotoxicity of anthracyclines. Front Cardiovasc Med. (2020) 7:26. 10.3389/fcvm.2020.00026 32258060 PMC7093379

[4] Pecoraro M Del Pizzo M Marzocco S Sorrentino R Ciccarelli M Iaccarino G. Inflammatory mediators in a short-time mouse model of doxorubicin-induced cardiotoxicity. Toxicol Appl Pharmacol. (2016) 293:44–52. 10.1016/j.taap.2016.01.006 26780402

[5] Tocchetti CG Cadeddu C Di Lisi D Femmino S Madonna R Mele D. From molecular mechanisms to clinical management of antineoplastic drug-induced cardiovascular toxicity: a translational overview. Antioxid Redox Signal. (2019) 30:2110–53. 10.1089/ars.2016.6930 28398124 PMC6529857

[6] Liu Y Liang Y Zheng B Chu L Ma D Wang H. Protective effects of crocetin on arsenic trioxide-induced hepatic injury: involvement of suppression in oxidative stress and inflammation through activation of Nrf2 signaling pathway in rats. Drug Des Devel Ther. (2020) 14:1921–31. 10.2147/DDDT.S247947 32546959 PMC7245440

[7] Nair AR Lee WK Smeets K Swennen Q Sanchez A Thevenod F. Glutathione and mitochondria determine acute defense responses and adaptive processes in cadmium-induced oxidative stress and toxicity of the kidney. Arch Toxicol. (2015) 89:2273–89. 10.1007/s00204-014-1401-9 25388156

[8] Xin Y Zhang S Gu L Liu S Gao H You Z. Electrocardiographic and biochemical evidence for the cardioprotective effect of antioxidants in acute doxorubicin-induced cardiotoxicity in the beagle dogs. Biol Pharm Bull. (2011) 34:1523–6. 10.1248/bpb.34.1523 21963490

[9] Mitry MA Edwards JG. Doxorubicin induced heart failure: Phenotype and molecular mechanisms. Int J Cardiol Heart Vasc. (2016) 10:17–24. 10.1016/j.ijcha.2015.11.004 27213178 PMC4871279

[10] Wang X Gao Y Tian Y Liu X Zhang G Wang Q. Integrative serum metabolomics and network analysis on mechanisms exploration of Ling-Gui-Zhu-Gan Decoction on doxorubicin-induced heart failure mice. J Ethnopharmacol. (2020) 250:112397. 10.1016/j.jep.2019.112397 31830550

[11] Ren D Li F Cao Q Gao A Ai Y Zhang J. Yangxin granules alleviate doxorubicin-induced cardiotoxicity by suppressing oxidative stress and apoptosis mediated by AKT/GSK3beta/beta-catenin signaling. J Int Med Res. (2020) 48:300060520945161. 10.1177/0300060520945161 32780664 PMC7425278

[12] Langer SW. Dexrazoxane for the treatment of chemotherapy-related side effects. Cancer Manag Res. (2014) 6:357–63. 10.2147/CMAR.S47238 25246808 PMC4168851

[13] Wang L Ma R Liu C Liu H Zhu R Guo S. *Salvia miltiorrhiza*: a potential red light to the development of cardiovascular diseases. Curr Pharm Des. (2017) 23:1077–97. 10.2174/1381612822666161010105242 27748194 PMC5421141

[14] Zhang J Zhang Q Liu G Zhang N. Therapeutic potentials and mechanisms of the Chinese traditional medicine danshensu. Eur J Pharmacol. (2019) 864:172710. 10.1016/j.ejphar.2019.172710 31586468

[15] Bao XY Zheng Q Tong Q Zhu PC Zhuang Z Zheng GQ. Danshensu for myocardial ischemic injury: Preclinical evidence and novel methodology of quality assessment tool. Front Pharmacol. (2018) 9:1445. 10.3389/fphar.2018.01445 30618743 PMC6297803

[16] Tang Y Wang M Le X Meng J Huang L Yu P. Antioxidant and cardioprotective effects of Danshensu (3-(3, 4-dihydroxyphenyl)-2-hydroxy-propanoic acid from *Salvia miltiorrhiza*) on isoproterenol-induced myocardial hypertrophy in rats. Phytomedicine. (2011) 18:1024–30. 10.1016/j.phymed.2011.05.007 21665454

[17] Yin Y Guan Y Duan J Wei G Zhu Y Quan W. Cardioprotective effect of Danshensu against myocardial ischemia/reperfusion injury and inhibits apoptosis of H9c2 cardiomyocytes via Akt and ERK1/2 phosphorylation. Eur J Pharmacol. (2013) 699:219–26. 10.1016/j.ejphar.2012.11.005 23200898

[18] Zhou X Chan SW Tseng HL Deng Y Hoi PM Choi PS. Danshensu is the major marker for the antioxidant and vasorelaxation effects of Danshen (*Salvia miltiorrhiza*) water-extracts produced by different heat water-extractions. Phytomedicine. (2012) 19:1263–9. 10.1016/j.phymed.2012.08.011 23026310

[19] Gao Y Zhang K Zhu F Wu Z Chu X Zhang X. Salvia miltiorrhiza (Danshen) inhibits L-type calcium current and attenuates calcium transient and contractility in rat ventricular myocytes. J Ethnopharmacol. (2014) 158(Pt A):397–403. 10.1016/j.jep.2014.10.049 25446591

[20] Gheorghiade M De Luca L Bonow RO. Neurohormonal inhibition in heart failure: insights from recent clinical trials. Am J Cardiol. (2005) 96:3L−9L. 10.1016/j.amjcard.2005.09.059 16399087

[21] Bin Jardan YA Ansari MA Raish M Alkharfy KM Ahad A Al-Jenoobi FI. Sinapic acid ameliorates oxidative stress, inflammation, and apoptosis in acute doxorubicin-induced cardiotoxicity via the NF-kappaB-mediated pathway. Biomed Res Int. (2020) 2020:3921796. 10.1155/2020/3921796 32258120 PMC7085847

[22] Hiona A Lee AS Nagendran J Xie X Connolly AJ Robbins RC. Pretreatment with angiotensin-converting enzyme inhibitor improves doxorubicin-induced cardiomyopathy via preservation of mitochondrial function. J Thorac Cardiovasc Surg. (2011) 142:396–403 e3. 10.1016/j.jtcvs.2010.07.097 21094500 PMC3173512

[23] Ibrahim MA Ashour OM Ibrahim YF El-Bitar HI Gomaa W Abdel-Rahim SR. Angiotensin-converting enzyme inhibition and angiotensin AT(1)-receptor antagonism equally improve doxorubicin-induced cardiotoxicity and nephrotoxicity. Pharmacol Res. (2009) 60:373–81. 10.1016/j.phrs.2009.05.007 19467331

[24] Chu X Zhang Y Xue Y Li Z Shi J Wang H. Crocin protects against cardiotoxicity induced by doxorubicin through TLR-2/NF-kappaB signal pathway *in vivo* and *in vitro*. Int Immunopharmacol. (2020) 84:106548. 10.1016/j.intimp.2020.106548 32388215

[25] Zhang R Zhu X Bai H Ning K. Network pharmacology databases for traditional Chinese medicine: review and assessment. Front Pharmacol. (2019) 10:123. 10.3389/fphar.2019.00123 30846939 PMC6393382

[26] Hopkins AL. Network pharmacology: the next paradigm in drug discovery. Nat Chem Biol. (2008) 4:682–90. 10.1038/nchembio.118 18936753

[27] Kibble M Saarinen N Tang J Wennerberg K Makela S Aittokallio T. Network pharmacology applications to map the unexplored target space and therapeutic potential of natural products. Nat Prod Rep. (2015) 32:1249–66. 10.1039/C5NP00005J 26030402

[28] Davis AP Grondin CJ Johnson RJ Sciaky D Wiegers J Wiegers TC. Comparative Toxicogenomics Database (CTD): update 2021. Nucleic Acids Res. (2021) 49:D1138–43. 10.1093/nar/gkaa891 33068428 PMC7779006

[29] Stelzer G Rosen N Plaschkes I Zimmerman S Twik M Fishilevich S. The GeneCards suite: From gene data mining to disease genome sequence analyses. Curr Protoc Bioinformatics. (2016) 54:1 30 1–33. 10.1002/cpbi.5 27322403

[30] Huang da W Sherman BT Lempicki RA. Systematic and integrative analysis of large gene lists using DAVID bioinformatics resources. Nat Protoc. (2009) 4:44–57. 10.1038/nprot.2008.211 19131956

[31] Huang da W Sherman BT Lempicki RA. Bioinformatics enrichment tools: paths toward the comprehensive functional analysis of large gene lists. Nucleic Acids Res. (2009) 37:1–13. 10.1093/nar/gkn923 19033363 PMC2615629

[32] Szklarczyk D Gable AL Lyon D Junge A Wyder S Huerta-Cepas J. STRING v11: protein-protein association networks with increased coverage, supporting functional discovery in genome-wide experimental datasets. Nucleic Acids Res. (2019) 47:D607–13. 10.1093/nar/gky1131 30476243 PMC6323986

[33] Zhang Y Zhang G Liang Y Wang H Wang Q Zhang Y. Potential mechanisms underlying the hepatic-protective effects of danshensu on iron overload mice. Biol Pharm Bull. (2020) 43:968–75. 10.1248/bpb.b19-01084 32475919

[34] Rochette L Guenancia C Gudjoncik A Hachet O Zeller M Cottin Y. Anthracyclines/trastuzumab: new aspects of cardiotoxicity and molecular mechanisms. Trends Pharmacol Sci. (2015) 36:326–48. 10.1016/j.tips.2015.03.005 25895646

[35] Li S Wang W Niu T Wang H Li B Shao L. Nrf2 deficiency exaggerates doxorubicin-induced cardiotoxicity and cardiac dysfunction. Oxid Med Cell Longev. (2014) 2014:748524. 10.1155/2014/748524 24895528 PMC4033424

[36] Wang LF Su SW Wang L Zhang GQ Zhang R Niu YJ. Tert-butylhydroquinone ameliorates doxorubicin-induced cardiotoxicity by activating Nrf2 and inducing the expression of its target genes. Am J Transl Res. (2015) 7:1724–35. Available online at: https://www.ncbi.nlm.nih.gov/pmc/articles/PMC4656753/26692920 PMC4656753

[37] Bellezza I Giambanco I Minelli A Donato R. Nrf2-Keap1 signaling in oxidative and reductive stress. Biochim Biophys Acta Mol Cell Res. (2018) 1865:721–33. 10.1016/j.bbamcr.2018.02.010 29499228

[38] Kansanen E Kuosmanen SM Leinonen H Levonen AL. The Keap1-Nrf2 pathway: Mechanisms of activation and dysregulation in cancer. Redox Biol. (2013) 1:45–9. 10.1016/j.redox.2012.10.001 24024136 PMC3757665

[39] Itoh K Wakabayashi N Katoh Y Ishii T Igarashi K Engel JD. Keap1 represses nuclear activation of antioxidant responsive elements by Nrf2 through binding to the amino-terminal Neh2 domain. Genes Dev. (1999) 13:76–86. 10.1101/gad.13.1.76 9887101 PMC316370

[40] Iranshahy M Iranshahi M Abtahi SR Karimi G. The role of nuclear factor erythroid 2-related factor 2 in hepatoprotective activity of natural products: A review. Food Chem Toxicol. (2018) 120:261–76. 10.1016/j.fct.2018.07.024 30009889

[41] Cui T Lai Y Janicki JS Wang X. Nuclear factor erythroid-2 related factor 2 (Nrf2)-mediated protein quality control in cardiomyocytes. Front Biosci. (2016) 21:4384. 10.2741/4384 26709769 PMC4723105

[42] Yarmohammadi F Rezaee R Karimi G. Natural compounds against doxorubicin-induced cardiotoxicity: a review on the involvement of Nrf2/ARE signaling pathway. Phytother Res. (2021) 35:1163–75. 10.1002/ptr.6882 32985744

[43] Sarkar S Mukherjee S Chattopadhyay A Bhattacharya S. Low dose of arsenic trioxide triggers oxidative stress in zebrafish brain: expression of antioxidant genes. Ecotoxicol Environ Saf. (2014) 107:1–8. 10.1016/j.ecoenv.2014.05.012 24905690

[44] Singh P Sharma R McElhanon K Allen CD Megyesi JK Benes H. Sulforaphane protects the heart from doxorubicin-induced toxicity. Free Radic Biol Med. (2015) 86:90–101. 10.1016/j.freeradbiomed.2015.05.028 26025579 PMC4554811

[45] Li J Ichikawa T Villacorta L Janicki JS Brower GL Yamamoto M. Nrf2 protects against maladaptive cardiac responses to hemodynamic stress. Arterioscler Thromb Vasc Biol. (2009) 29:1843–50. 10.1161/ATVBAHA.109.189480 19592468 PMC12952473

[46] Razmaraii N Babaei H Mohajjel Nayebi A Assadnassab G Ashrafi Helan J Azarmi Y. Crocin treatment prevents doxorubicin-induced cardiotoxicity in rats. Life Sci. (2016) 157:145–51. 10.1016/j.lfs.2016.06.012 27297631

[47] Cove-Smith L Woodhouse N Hargreaves A Kirk J Smith S Price SA. An integrated characterization of serological, pathological, and functional events in doxorubicin-induced cardiotoxicity. Toxicol Sci. (2014) 140:3–15. 10.1093/toxsci/kfu057 24675088

[48] Alam MF Khan G Safhi MM Alshahrani S Siddiqui R Sivagurunathan Moni S. Thymoquinone ameliorates doxorubicin-induced cardiotoxicity in Swiss albino mice by modulating oxidative damage and cellular inflammation. Cardiol Res Pract. (2018) 2018:1483041. 10.1155/2018/1483041 29805796 PMC5901949

[49] Putri H Nagadi S Larasati YA Wulandari N Hermawan A Nugroho AE. Cardioprotective and hepatoprotective effects of Citrus hystrix peels extract on rats model. Asian Pac J Trop Biomed. (2013) 3:371–5. 10.1016/S2221-1691(13)60079-9 23646300 PMC3642446

[50] Renu K Abilash VG Tirupathi Pichiah PB Arunachalam S. Molecular mechanism of doxorubicin-induced cardiomyopathy - an update. Eur J Pharmacol. (2018) 818:241–53. 10.1016/j.ejphar.2017.10.043 29074412

[51] Chichuk TV Strashkevich IA Klebanov GI. Free radical mechanisms of low-intensive laser radiation. Vestn Ross Akad Med Nauk. (1999) 2:27–32. 10204020

[52] Thangapandiyan S Miltonprabu S. Epigallocatechin gallate effectively ameliorates fluoride-induced oxidative stress and DNA damage in the liver of rats. Can J Physiol Pharmacol. (2013) 91:528–37. 10.1139/cjpp-2012-0347 23826622

[53] Zhang J Cui L Han X Zhang Y Zhang X Chu X. Protective effects of tannic acid on acute doxorubicin-induced cardiotoxicity: Involvement of suppression in oxidative stress, inflammation, and apoptosis. Biomed Pharmacother. (2017) 93:1253–60. 10.1016/j.biopha.2017.07.051 28738542

[54] Turillazzi E Neri M Cerretani D Cantatore S Frati P Moltoni L. Lipid peroxidation and apoptotic response in rat brain areas induced by long-term administration of nandrolone: the mutual crosstalk between ROS and NF-κB. J Cell Mol Med. (2016) 20:601–12. 10.1111/jcmm.12748 26828721 PMC5125979

[55] Rahman I. Oxidative stress, transcription factors and chromatin remodelling in lung inflammation. Biochem Pharmacol. (2002) 64:935–42. 10.1016/S0006-2952(02)01153-X 12213589

[56] Abd El-Aziz TA Mohamed RH Pasha HF Abdel-Aziz HR. Catechin protects against oxidative stress and inflammatory-mediated cardiotoxicity in adriamycin-treated rats. Clin Exp Med. (2012) 12:233–40. 10.1007/s10238-011-0165-2 22080234

[57] Hettmann T DiDonato J Karin M Leiden JM. An essential role for nuclear factor kappaB in promoting double positive thymocyte apoptosis. J Exp Med. (1999) 189:145–58. 10.1084/jem.189.1.145 9874571 PMC1887697

[58] Gilleron M Marechal X Montaigne D Franczak J Neviere R Lancel S. oxidases participate to doxorubicin-induced cardiac myocyte apoptosis. Biochem Biophys Res Commun. (2009) 388:727–31. 10.1016/j.bbrc.2009.08.085 19699179

[59] Wallace KB Sardao VA Oliveira PJ. Mitochondrial determinants of doxorubicin-induced cardiomyopathy. Circ Res. (2020) 126:926–41. 10.1161/CIRCRESAHA.119.314681 32213135 PMC7121924

[60] Adams JM Cory S. The BCL-2 arbiters of apoptosis and their growing role as cancer targets. Cell Death Differ. (2018) 25:27–36. 10.1038/cdd.2017.161 29099483 PMC5729526

[61] Pham TN Marion M Denizeau F Jumarie C. Cadmium-induced apoptosis in rat hepatocytes does not necessarily involve caspase-dependent pathways. Toxicol Vitro. (2006) 20:1331–42. 10.1016/j.tiv.2006.05.005 16809017

[62] Hengartner MO. The biochemistry of apoptosis. Nature. (2000) 407:770–6. 10.1038/35037710 11048727

[63] Danial NN Korsmeyer SJ. Cell death: critical control points. Cell. (2004) 116:205–19. 10.1016/S0092-8674(04)00046-7 14744432

[64] Brentnall M Rodriguez-Menocal L De Guevara RL Cepero E Boise LH. Caspase-9, caspase-3 and caspase-7 have distinct roles during intrinsic apoptosis. BMC Cell Biol. (2013) 14:32. 10.1186/1471-2121-14-32 23834359 PMC3710246

[65] Chang HY Yang X. Proteases for cell suicide: functions and regulation of caspases. Microbiol Mol Biol Rev. (2000) 64:821–46. 10.1128/MMBR.64.4.821-846.2000 11104820 PMC99015

[66] Elmore S. Apoptosis: a review of programmed cell death. Toxicol Pathol. (2007) 35:495–516. 10.1080/01926230701320337 17562483 PMC2117903

[67] He H Luo Y Qiao Y Zhang Z Yin D Yao J. Curcumin attenuates doxorubicin-induced cardiotoxicity via suppressing oxidative stress and preventing mitochondrial dysfunction mediated by 14-3-3gamma. Food Funct. (2018) 9:4404–18. 10.1039/C8FO00466H 30063064

[68] Hu C Zhang X Song P Yuan YP Kong CY Wu HM. Meteorin-like protein attenuates doxorubicin-induced cardiotoxicity via activating cAMP/PKA/SIRT1 pathway. Redox Biol. (2020) 37:101747. 10.1016/j.redox.2020.101747 33045622 PMC7558217

